# Correlation between cognitive functions and central auditory processing in adolescents with non-cholesteatomatous chronic otitis media

**DOI:** 10.1590/1980-57642018dn12-030013

**Published:** 2018

**Authors:** Márcia Salgado Machado, Adriane Ribeiro Teixeira, Sady Selaimen da Costa

**Affiliations:** 1 Speech Therapist, Ph.D. Adjunct Professor, Department of Speech Pathology, Federal University of Health Sciences of Porto Alegre, Porto Alegre, RS, Brazil.; 2 Speech Therapist, Ph.D., Associate Professor, Department of Health and Human Communication, Federal University of Rio Grande do Sul, Porto Alegre, RS, Brazil..; 3 Otorhinolaryngologist, Ph.D., Associate Professor, Department of Ophthalmology and Otorhinolaryngology, Federal University of Rio Grande do Sul, Porto Alegre, RS, Brazil.

**Keywords:** auditory perception, adolescent, attention, memory, executive function, percepção auditiva, adolescente, atenção, memória, função executiva

## Abstract

Auditory deprivation associated with early otitis media with effusion has been considered a risk factor for central auditory processing (CAP), as well as for the development of a number of cognitive functions.

**Objective:**

To study the neuropsychological functions of attention, working memory and executive function in adolescents with and without non-cholesteatomatous chronic otitis media (NCCOM) and analyze their interrelationships with the behavioral evaluation of CAP.

**Methods:**

Sixty-eight adolescents were recruited, 34 were diagnosed with NCCOM (study group - SG), and 34 had no otological history (control group - CG). The Neupsilin Brief Neuropsychological Assessment Instrument was used. CAP was assessed by: Masking Level Difference, Synthetic Sentence Identification, Random Gap Detection Test, Duration Pattern Sequence Test and Dichotic Digits Test.

**Results:**

The results of Neupsilin showed lower scores in the study group when compared to the control group on the following tests: digit sequence repetition, ascending digit ordering, auditory sentence span, and phonemic verbal fluency. An association was found between central auditory processing tests and Neupsilin subtests.

**Conclusion:**

The effects of NCCOM on attention, memory and executive function related to central auditory processing disorder in adolescents seem to be enhanced by the severity of the disease.

## Resumo

A privação auditiva associada à otite média precoce com efusão tem sido considerada um fator de risco para o processamento auditivo central (CAP), bem como para o desenvolvimento de diversas funções cognitivas.

**Objetivo:**

Estudar as funções neuropsicológicas da atenção, memória de trabalho e função executiva em adolescentes com e sem otite média crônica não-colesteatomatosa (NCCOM) e analisar suas inter-relações com a avaliação comportamental da PAC.

**Métodos:**

Sessenta e oito adolescentes foram recrutados, 34 foram diagnosticados com NCCOM (grupo de estudo - GE) e 34 não tinham história otológica (grupo controle - GC). Utilizou-se o Instrumento de Avaliação Neuropsicológica Breve de Neupsilin. O CAP foi avaliado por: Diferença de Nível de Mascaramento, Identificação de Frase Sintética, Teste de Detecção de Intervalo Aleatório, Teste de Sequência de Padrão de Duração e Teste de Dígitos Dicóticos.

**Resultados:**

Os resultados de Neupsilin mostraram menores escores no grupo de estudo quando comparados ao grupo controle nos seguintes testes: repetição de sequência de dígitos, ordenação de dígitos ascendentes, extensão de sentenças auditivas e fluência verbal fonêmica. Foi encontrada uma associação entre os testes do processamento auditivo central e os subtestes de Neupsilin.

**Conclusão:**

Os efeitos do NCCOM na atenção, memória e função executiva relacionados ao transtorno do processamento auditivo central em adolescentes parecem ser potencializados pela gravidade da doença.

Central auditory processing consists of a summation of specific skills necessary for the individual to understand what they hear.^1^ However, central auditory processing is also shared with other cognitive functions,^2^ such as attention, memory, language, executive functions, among others.^3^ These functions share actions with specific auditory structures of the auditory central nervous system during the processing of auditory information.^4^ Therefore, it is necessary to study and include neuroscience on the understanding of the interfaces between central auditory processing and cognitive functions.^5^

The literature indicates evidence of attention function impairment in individuals with a history of otitis media,^6^^,^ 7 as well as likely associations between working memory and hearing in a noisy environment.^8^^-^ 10 In addition, a study^11^ observed that the executive function shares cognitive mechanisms underlying auditory skills.

Thus, there is great interest in the interrelationships between auditory and cognitive abilities.^12^ In this regard, auditory deprivation associated with early otitis media with effusion has been considered a risk factor not only for central auditory processing but also for the development of some cognitive functions.^13^ A number of studies have investigated the effects of early otitis media with effusion on cognitive development,^6^^,^ 7 ^,^ 14 ^-^ 17 but no studies on subjects with chronic otitis media were found.

The aim of this study was to investigate the neuropsychological functions of attention, working memory and executive function in adolescents with and without NCCOM and to analyze their interrelationships with the behavioral evaluation of central auditory processing and with the variables: gender, age, education repetition, age of onset of otitis media, unilaterality or bilaterality of auditory alteration, maternal education and family income.

## METHODS

This study consisted of a controlled cross-sectional, observational study for which 68 adolescents aged 12 - 18 years^18^ were recruited, 34 of whom had non-cholesteatomatous chronic otitis media (study group - SG), while 34 had no otological history (control group - CG).

For the control group, the following inclusion criteria were applied: adolescents from public schools, with no history of otitis media, with normal audiological assessment and typical overall development. The study group consisted of adolescents from public schools with a diagnosis of unilateral or bilateral NCCOM and average of auditory thresholds (obtained from tonal audiometry) at the frequencies of 500, 1000, 2000 and 4000 Hz ≤ 40 dB HL in the affected ear(s).

For both groups, the following exclusion criteria were considered: presence of mental, neurological disorders or genetic syndromes, being left-handed, having a history of formal music education and presenting with other risk factors for hearing loss. The information about these criteria was collected by analyzing the clinical records of each patient (study group) or through anamnesis with their parents (control group).

The subjects who agreed to participate in the study underwent the following procedures: anamnesis; audiological assessment (pure tone audiometry and speech audiometry); central auditory processing behavioral evaluation battery; and neuropsychological assessment of attention, working memory and executive functions (verbal fluency). All proposed assessments were performed by duly trained researchers experienced in performing the tests described.

The test battery assessing auditory processing was composed of the following tests: MLD (Masking Level Difference), SSI (Synthetic Sentence Identification), RGDT (Random Gap Detection Test), DPS (Duration Pattern Sequence test) and DDT (Dichotic Digits Test), according to the recommendations of the Academia Brasileira de Audiologia^19^ related to the minimum protocol for CAP behavioral evaluation. MLD was performed at 70dB intensity in the ear (s) with normal auditory thresholds (mean square ≤25dB) or 50dBLS (decibel level of sensation) in the ear (s) with altered auditory thresholds (mean square >25dB). The SSI was applied at a 40dBLS intensity in the main message, and the intensity of the ipsilateral competitive message was performed under two signal-to-noise ratio conditions (0 and -15dB). RGDT, DPS and DDT were performed at an intensity level of 50dBLS in each ear. The intensity of application of the tests (dBLS) was calculated from the mean airway auditory thresholds of 500, 1000 and 2000 Hz frequencies of each ear.

To analyze the results obtained on CAP tests among adolescents in the study group with unilateral and bilateral conductive alterations, this group was subdivided into two groups: unilateral air gap group (obtained by means of threshold tonal audiometry airway and bone pathway) (UG) and bilateral gap group (BG). It should be noted that this differential was considered even in individuals who did not present auditory thresholds indicative of hearing loss, being characterized as a conductive component caused by NCCOM, which did not necessarily induce classification of hearing loss degree. The parameter used to characterize the presence of gap was a differential ≥15dB between the tonal thresholds obtained by airway and bone pathway at the frequencies of 500 to 4000 Hz.^20^ In order to evaluate the neuropsychological aspects selected for this study, the Neupsilin Brief Neuropsychological Assessment Instrument^21^^,^ 22 was applied according to the instructions described in the instrument manual. However, only the following subtests were conducted: attention (reverse counting and digit sequence repetition), working memory (ascending digit ordering and auditory sentence span), and executive function (verbal fluency test).

On the reverse counting test, the maximum score was 20 points, each point representing a correct answer. On the digit sequence repetition test, the maximum score was seven points. Thus, the total score for the attention tests was 27 points.

On the ascending digit ordering task, the maximum score was ten points, while, for auditory sentence span, the maximum score was 28 points.

On the phonemic verbal fluency test, the score was established by the number of items uttered during the time stipulated for the test.

It is important to clarify that the criteria of normality set forth in the test manual had to be adapted to the study sample, since the mean and standard deviation values available in the manual are presented according to grades (7^th^ and 8^th^ grades in elementary school and 1^st^, 2^nd^ and 3^rd^ grades in high school). However, some subjects in this study, although having the minimum age for administration of the test (12 years), were not yet in the 7^th^ grade (due to school repetition). As a result the parameter indicated for the 7^th^ grade was used, because, due to the chronological aspect, it should be the current grade for these subjects. Furthermore, by virtue of the change in the number of grades in elementary schools in the country (which rose from eight to nine years), subjects who attended the 9^th^ grade were included in the parameters of normality for the 8^th^ grade, since the test manual does not provide parameters for this grade.

The sample size was calculated using the WINPEPI software, version 11.43. For a significance level of 5%, 90% power, and a minimum effect size of 0.8 standard deviations between groups, a minimum total of 33 individuals were obtained per group, totaling 66 individuals.

The statistical analysis of data was performed as follows: the qualitative variables were expressed as absolute and relative frequencies, Pearson’s Chi-square test or Fisher’s exact test were used for the comparison of proportions, and the Pearson (symmetrical distribution) or Spearman (asymmetric distribution) correlation coefficients were applied to evaluate the association between test results. The level of significance was set at 5% (p≤0.05), and analyses were carried out using the SPSS software version 21.0.

This study was approved by the Research Ethics Committee of the Institution of the study, with approval protocol 41689215.7.0000.5327.

## RESULTS

The characteristics of the sample studied can be seen in Table 1.

**Table 1:** Sample characteristics.

Variables		Study group (n=34)	Control group (n=34)	P
Age (years) – mean±SD		14.9±2.1	15.1±2.1	
Gender n (%) – mean±SD	Male	22 (64.7)	22 (64.7)	
	Female	12 (35.3)	12 (35.3)	
Years of education – mean±SD		8.8±1.9	9.3±2.3	
AW – mean±SD	RE	21.2±10.8	5.9±3.9	<0.001*
	LE	21.2±11.4	5.7±3.4	<0.001*
Air bone gap mean – mean±SD	RE	19.3±9.6	---	
	LE	17.7±9.0	---	
CAPD diagnosis– n (%)		34 (100)	---	

n: number; SD: standard deviation; AW: airway.

* p≤0.05 (level of statistical significance).

Table 2 shows the results for the Neupsilin subtests conducted in the control and study groups.

**Table 2:** Neuropsychological test results.

Variables		Study group (n=34)	Control group (n=34)	P
Attention – Reverse counting	Normal	29 (85.3)	34 (100)	0.053
	Abnormal	5 (14.7)	0 (0.0)	
Attention – Digit sequence repetition	Normal	27 (79.4)	34 (100)	0.011*
	Abnormal	7 (20.6)	0 (0.0)	
Attention – Total score	Normal	30 (88.2)	34 (100)	0.114
	Abnormal	4 (11.8)	0 (0.0)	
Working memory – Ascending digit ordering	Normal	28 (82.4)	34 (100)	0.025*
	Abnormal	6 (17.6)	0 (0.0)	
Working memory – Auditory sentence span	Normal	25 (73.5)	34 (100)	0.002*
	Abnormal	9 (26.5)	0 (0.0)	
Executive function – Verbal Fluency Test	Normal	23 (67.6)	34 (100)	0.001*
	Abnormal	11 (32.4)	0 (0.0)	

n: number;

* p≤0.05 (level of statistical significance).

Table 3 shows the association between central auditory processing tests and neuropsychological tests using the Spearman correlation coefficient.

**Table 3:** Association between CAP tests and neuropsychological tests using the Spearman correlation coefficient.

Variables		Neupsilin results					
		Reverse counting	Digit sequence repetition	Attention Total score	Ascending digit ordering	Auditory sentence span	Verbal Fluency Test
SSI 0	RE	0.039	0.241	0.090	0.168	0.059	0.326
	LE	0.097	0.173	0.159	0.477**	–0.218	0.342*
SSI –15	RE	0.095	0.292	0.119	0.133	–0.087	0.219
	LE	0.104	0.219	0.180	0.221	–0.205	0.392*
DDT	RE	0.346*	0.428*	0.376*	0.235	0.125	0.188
	LE	0.431*	0.346*	0.434*	0.025	0.018	0.354*
DPS		0.390*	0.056	0.322	0.130	0.072	0.260
MLD		0.082	0.128	0.000	–0.084	0.200	0.039
RGDT		–0.353*	–0.298	–0.285	0.047	–0.205	–0.026

* p<0.05;

** p<0.01;

RE: right ear; LE: left ear; SSI: Synthetic Sentence Identification; DDT: Dichotic Digits Test; DPS: Duration Pattern Sequence; MLD: Masking Level Difference; RGDT: Random Gap Detection Test. *p≤0.05 (level of statistical significance).

**Table 4:** Central auditory processing tests in SG subjects with unilateral and bilateral conductive loss.

Variables		UG (n=17)	BG (n=16)	P
SSI 0 – mean±SD	RE	52.9±9.9	55.0±19.7	0.710
	LE	52.9±10.5	55.6±18.6	0.610
SSI 15 – mean±SD	RE	25.9±13.7	33.1±17.8	0.198
	LE	27.0±10.5	37.5±18.1	0.055
DDT – mean±SD	RE	94.7±5.5	97.8±2.7	0.049*
	LE	96.6±3.5	98.9±1.8	0.027*
DPS – mean±SD		43.9±23.3	46.0±21.5	0.784
MLD – mean±SD		8.9±4.1	9.3±3.0	0.807
RGDT – mean±SD		12.9±6.5	15.3±6.4	0.287

UG: unilateral group; BG; bilateral group; n: number; SD: standard deviation; RE: right ear; LE: left ear; SSI: Synthetic Sentence Identification; DDT: Dichotic Digits Test; DPS: Duration Pattern Sequence; MLD: Masking Level Difference; RGDT: Random Gap Detection Test.

* p≤0.05 (level of statistical significance).

No association was found between the results for the Neupsilin subtests when compared for gender (p>0.20), age (p> 0.05), school repetition (p>0.2), age at otitis media onset (p>0.3), maternal education (p>0.4) and family income (p>0.05). All SG individuals had alterations in at least two physiological mechanisms of CAP, demonstrating 100% of central auditory processing disorder (CAPD) in adolescents with NCCOM evaluated. Comparing the results obtained between the groups with unilateral and bilateral conductive changes, significantly worse scores were observed in the UG when compared to the BG in DDT in the right ear and in the left ear (p =0.049 and p =0.027, respectively). Although the other tests showed no statistical difference, it is noteworthy that the scores were numerically worse in the UG on the SSI, MLD and DPS tests. It is important to note that the BG group had better results on the RGDT test, since lower absolute value represents a better result on this test.

## DISCUSSION

This study showed alterations and correlations of some neuropsychological functions with the CAP of the adolescents evaluated. In addition, a change in the CAP of adolescents with NCCOM was observed, with a more significant impact on subjects who had a unilateral conductive component.

The Neupsilin results showed lower scores in the study group when compared to the control group on the following tests: digit sequence repetition (attention), ascending digit ordering (working memory), auditory sentence span (working memory), and phonemic verbal fluency (executive function). An association was found between the DD, DPS and RGDT tests and the reverse counting test (attention), and there was also an association between the DDT and the digit sequence repetition (attention) subtest and the overall score for the attention function and phonemic verbal fluency (executive function). In addition, the SSI was associated with working memory (ascending digit ordering subtest) and executive function (phonemic verbal fluency).

Regarding the changes on the attention test (digit sequence repetition), a likely relationship between attention and CAP is believed to exist, and has previously been documented in some studies conducted in individuals with a history of otitis media.^6^^,^ 7 ^,^ 23 The study by Haapala et al.,^23^ who investigated the consequences of recurrent acute otitis media on the involuntary auditory attention of two-year-old infants through late-latency evoked potentials is noteworthy In the study, the authors detected an immature control of attentional shift in children with recurrent acute otitis media and associated it with the typical distraction behavior of these children. Additionally, they also identified increased MMN (mismatch negativity) latency, which suggests a delay in attentional reorienting for resumption of an ongoing activity in the sample studied. Therefore, when exposed to disturbing sounds, the subjects with a history positive for otitis media showed atypical neural organization in the neural mechanisms of involuntary attention.^23^

It should be noted that, in this study, two subtests were used to assess the attention function. However, only one of them (digit sequence repetition) showed a significant difference between the study and control groups. This is believed to have occurred due to the ease of the reverse counting test, while the digit sequence repetition test is more complex and, consequently, poses a higher degree of attentional requirement.

Concerning the differences observed between the groups on the working memory assessment subtests, it should be noted that, when there is a deficit in auditory input processing, so much effort is spent on understanding the auditory information that little energy is left for it to be remembered.^24^ Thus, in cases of slow auditory input processing, the information presented at the beginning of the communication cannot be kept in the working memory at the point when the last part of the message is processed and, as a result, the whole message may be “forgotten” or not fully understood.^22^ Additionally, this interrelationship has already been described in the literature^8^^-^ 10 ^,^ 25 as these are tasks performed by neighboring brain regions.^8^ An example of this is the hippocampus in its lower medial portion of the anterior temporal lobe. On the other hand, a study^26^ found no relationship between speech perception in the presence of noise and working memory in schoolchildren when investigating the biological and cognitive processes involved in speech perception in a noisy environment during early childhood. However, the author stressed the complexity of speech perception in the presence of noise, which consists of a complex task requiring the simultaneous integration of sensory processing, attention, memory, and linguistic knowledge.^26^

With respect to the difference observed between groups on the subtest assessing phonemic verbal fluency, this is an expected result, since verbal fluency is essential for individuals to be able to present complex and interconnected behaviors, both in social/communicative interaction contexts and in complex cognitive situations that require greater mental control and reasoning^1^ as in CAP. Additionally, in the study by Prando et al.,^11^ the executive function was found to be one of the tasks that was least dissociated from CAP tasks, showing that this function shares cognitive mechanisms underlying the auditory abilities and therefore supports the findings in this study.

Regarding the associations identified between the Neupsilin subtests and the CAP behavioral evaluation tests, a previous study^11^ can help elucidate the results observed in the present study. The authors demonstrated strong correlations between the performance of adolescents on tests assessing CAP and tasks assessing neuropsychological skills such as attention and working memory, explained by the sharing of underlying cognitive abilities between them.

In this regard, from a cognitive neuroscience perspective, there are few (if any) fully compartmentalized areas in the brain that are responsible for only one sensory modality,^11^ since brain organization is not modular.^27^ According to the American Speech-Language-Hearing Association,^28^ there are indications that the processing of sensory information is interdependent and integrated into cognitive domains that include attention and memory. Hence, the correlations observed in this study between the CAP tests and Neupsilin tasks can be understood.

Prando et al.^11^ observed a correlation between the SSI and an attention task, which was justified by the authors as a result of the high attention demand required to perform the task. However, this correlation was not observed in the present study, but instead a relationship of the SSI with the working memory and verbal fluency tasks was demonstrated. These results are similar to other studies, which observed correlations between speech perception in a noisy environment and working memory.^8^^-^ 10 ^,^ 25

The other associations observed in this study were not found in the literature. However, it is believed that these occurred due to the overlapping of brain areas involved in performing general auditory and cognitive tasks,^29^ which maintained a relationship of interdependence between these processes.^24^ Likewise, Prando et al.,^11^ stressed that there is no causal relationship between neuropsychological function deficits and auditory perception deficits; therefore, the CAP evaluation should be performed and analyzed so as to complement the neuropsychological assessment.

The results presented in this study confirm the findings in the literature related to the correlations observed between CAP skills and attention and memory functions.^11^^,^ 24 However, studies involving these correlations are still scarce,^11^ which prevents this issue from being fully elucidated.

As regards NCCOM, specifically, it is important to note that the extent of the auditory deprivation effect required to cause a cognitive alteration in humans is unknown,^30^ since this is a dynamic disease^31^ that should be regarded as an event that is part of a continuous disease process.^32^ Regarding the effects of unilateral conductive hearing loss, Polley et al (2013)^33^ emphasized that attenuation and delay in sound conduction would cause distortions of acoustic cues used for sound localization and other aspects of binaural hearing. Williams and Jacobs (2009)^34^ also pointed out that the unilateral conductive hearing loss present in otitis media can cause asymmetry in the auditory levels of the ears, which justifies the results observed in this study in relation to the adolescents with unilateral and bilateral conductive alterations associated with the NCCOM.

In addition, the auditory deprivation arising from NCCOM does not occur only in the developmental period of the central auditory nervous system, but accompanies the individual in a fluctuating or permanent manner over long periods.

Therefore, the role of otitis media in the development of neuropsychological aspects still divides professionals with different views.^13^^,^ 34 However, it is accepted in the literature that an early history of chronic otitis media with effusion is associated with a higher incidence of learning difficulties, language deficits and attention disorders.^24^ The hypothesis is that this occurs because the reception and analysis of sound characteristics (such as frequency, intensity, and duration) serve as a basis for constructing language, especially with regard to the development of the memory of sound patterns in language.^35^

However, it is important to stress that no similar studies on subjects with chronic otitis media were found, where related studies tend to focus on early otitis media with effusion. Therefore, it can be assumed that, in NCCOM, the effects on CAP and cognition are probably enhanced by the severity of the disease.

It should also be noted that, in this study, the complete neuropsychological evaluation proposed in Neupsilin was not performed, since previous studies have linked CAP to attention and working memory functions. However, we suggest that further studies be conducted using the complete assessment battery of the instrument to allow the analysis of other hypotheses involving other cognitive functions.

Given that the data obtained reinforces the hypothesis that the central auditory function involves far more than a central nervous system roadmap to the auditory portion,^11^ this study raises questions about changes in neuropsychological functions in subjects with NCCOM and CAPD, which should be addressed and investigated in further studies.

This study found that the neuropsychological functions of attention, working memory and executive function (phonemic verbal fluency) showed alterations in adolescents with NCCOM when compared with the control group. In addition, associations were found between the results for the subtests assessing neuropsychological functions and the tests used for the behavioral evaluation of central auditory processing in adolescents with NCCOM. Thus, the effects of NCCOM on the tested attention, memory and executive functions relating to the central auditory processing disorder in adolescents seem to be enhanced by the severity of the disease.
